# Emerging Roles of Aryl Hydrocarbon Receptors in the Altered Clearance of Drugs during Chronic Kidney Disease

**DOI:** 10.3390/toxins11040209

**Published:** 2019-04-07

**Authors:** Tacy Santana Machado, Claire Cerini, Stéphane Burtey

**Affiliations:** 1 CAPES Foundation, Ministry of Education of Brazil, Brasília DF 70040-020, Brazil; tacysantana@hotmail.com; 2 Aix Marseille Université, INSERM, C2VN, 13005 Marseille, France; stephane.burtey@univ-amu.fr; 3 APHM, Conception Hospital, Centre de Néphrologie et Transplantation Rénale, 13005 Marseille, France; 4 European Uraemic Toxin Working Group of ESAO, endorsed by ERA-EDTA [EUTox], 3500 Krems, Austria

**Keywords:** AhR, uremic toxins, drug clearance

## Abstract

Chronic kidney disease (CKD) is a major public health problem, since 300,000,000 people in the world display a glomerular filtration rate (GFR) below 60 mL/min/1.73m^2^. Patients with CKD have high rates of complications and comorbidities. Thus, they require the prescription of numerous medications, making the management of patients very complex. The prescription of numerous drugs associated with an altered renal- and non-renal clearance makes dose adjustment challenging in these patients, with frequent drug-related adverse events. However, the mechanisms involved in this abnormal drug clearance during CKD are not still well identified. We propose here that the transcription factor, aryl hydrocarbon receptor, which is the cellular receptor for indolic uremic toxins, could worsen the metabolism and the excretion of drugs in CKD patients.

## 1. Chronic Kidney Disease

Chronic kidney disease (CKD) incidence has increased in recent years, and the global prevalence is estimated to be 8% to 16% [1,2]. The global prevalence of CKD is about 200 million people [3]. In the end of 2009, in the United Kingdom, the average prevalence was 794 per million population [4]. In India, the average age-adjusted incidence rate between the years 2002 to 2005, was of 229 per million population [5]. In the United States, approximately 20 million people have CKD, and the estimated cost for their care (CKD and CKD-related comorbidities) is $26,000 per patient [6]. This high prevalence of CKD makes it a broad public health problem. CKD is characterized by kidney damage, including structural or functional impairments maintained for at least 3 months. CKD is mainly defined as an increase in the urinary excretion of albumin and a decrease in glomerular filtration rate (GFR) [7]. Considering the estimated levels of GFR, CKD is classified intofive stages. Individuals with normal renal function are included in stage 1 and have a GFR equal to or greater than 90 mL/min/1.73 m^2^. A persistent GFR below 60 mL/min/1.73 m^2^ (stage 3) indicates a moderate loss of kidney function. In stage 5, GFR is lower than 15 mL/min/1.73m^2^, due to the kidney failure; these patients require chronic dialysis treatment or kidney transplantation (National Kidney Foundation, https://www.kidney.org, 2002).

Cardiovascular disease (CVD) is the main cause of death in patients with CKD. The cardiovascular mortality rate is 10 to 30 times higher than in the general population [8,9]. Kidney function impairment is associated with left ventricular hypertrophy [10], increased arterial stiffness and calcification [11,12,13], arterial thrombosis [14] and hemostatic disorders [15], and endothelial injury and dysfunction [16,17,18,19]. Traditional risk factors, such as diabetes, hypertension, dyslipidemia, and tobacco use are not enough to explain this high mortality in CKD patients [9,20]. Thus, specific risk factors associated with CKD were proposed to play a major role in cardiovascular mortality, such as uremic toxins [21].

Since patients with CKD have high rates of complications and comorbidities, there is a relevant impact on medication administration. Specific and related complications of CKD increase the need fordrug intake, so the dose adequacy is difficult to identify and drug side effects are more frequent [22,23,24]. All of the kinds of drugs used for CKD treatment and CKD-related diseases are listed in Table 1. Concomitant intake of assorted drugs can affect the efficacy of drug therapy due to drug–drug interactions. Drugs’ efficacy can also be affected by interindividual variations, such as genetic polymorphisms, epigenetic influences, microbiota, sex, age and disease states [25,26,27,28,29]. Understanding factors that can modify the metabolism of drugs is essential to improve the accuracy of prescriptionsforpatients. Furthermore, it is well known that renal impairments modify drug disposition and clearance in patients with CKD. Indeed, subjects exhibiting a smaller kidney size, a low number of functioning nephrons, and a decreased renal blood flow have a reduced capacity to eliminate substrates. To avoid toxicity, adverse effects, and poor outcomes, methods have been developed to estimate the real required dose of a drug. For example, the “Dettli method”considers the kidney elimination rate and the patient creatinine level. It assumes that the renal excretion reduces linearly in relation to this marker of renal function, whereas the non-renal clearance remains unchangeable [30,31]. For a long time, it was assumed unnecessary to adjust the dosage of drugs that are mainly metabolized and eliminated by the liver. However, during CKD, even drugs with a non-renal elimination require dosage adjustments to individualize therapy [32,33]. This is presumably a result of changes in drug pharmacokinetics, including the expression and activity of drug-metabolizing enzymes (DME) and drug transporters.

## 2. Drug Metabolism

Normally, the human body is capable of metabolizing and clear xenobiotics. Whether xenobiotics primarily belong to dietary compounds including plant and fungal-derived metabolites, they now include environmental pollutants and drugs. Most xenobiotics undergo two main phases, I and II, during their process of transformation, before their elimination from the body [34,35]. The first phase consists mainly of reactions of oxidation, reduction, hydrolysis, and hydration. It consists ofthe addition of a functional group (–OH, –COOH, –SH, –O–, or NH_2_), and it plays an important role in controlling the drug’s biological activity. Reactions in phase I are mainly led by the cytochrome P450 (CYP) superfamily of microsomal enzymes. These DME are expressed abundantly in the liver, gastrointestinal tract, kidney, and lung [36]. CYPs, especially CYP1A1/2 and CYP1B1, possess many binding sites, so they can metabolize several substrates with different structures. Besides drugs, CYPs can also interact with dietary compounds, such as caffeine, theophylline, mushrooms, naringin, and furanocoumarins, which are components found in grapefruit juice. CYPs can also interact with endogenous substrates like cholesterol, steroids, and bile acid. For example, the widely-consumed caffeine and theophylline undergo a complex metabolism, in which they are mainly transformed by CAP1A2 and to a lesser extent by CYP3A4 and CYP2E1 [37,38]. CYP2C9 is also involved in caffeine 7-*N*-demethylation and in the oxidation of steroids, such as progesterone and testosterone, as well as all-trans-retinoic acid [39]. Grapefruit juice at 25% inhibits the activities of CYP3A4, CYP2D6, and CYP2C9, and one of its compounds, bergamottin, inhibits the activities of CYP1A2, CYP2C9, CYP2C19, and CYP2D6 [40]. In the classic pathway of bile acid synthesis, cholesterol is converted to 7α-hydroxycholesterol parCYP7A1. Drug–drug interactions can also induce or inhibit CYP activity, influencing the metabolism as well as the toxicity and efficacy of one or both drugs [41]. In phase II, drugs and their metabolites are subjected to conjugative reactions, such as glucuronidation, methylation, sulfation, and acetylation (–OH, –NH_2_, –CH_3_,–SO_3_H_,_ or –CH_3_CO). The aim of this phase is to convert phase I substrates and their potentially toxic derivates into molecules that are less hydrophobic and that have an increased molecular weight. This results in the production of metabolites that are more easily excreted through the urine or the bile [41]. However, occasionally, this step produces biological active intermediates, resulting in an increased activity or toxicity [40,42]. Phase I and II enzymes play an important role preparing xenobiotics for the third phase: their elimination through the action of cellular transporters [43].

Transporters play a major role in drug pharmacokinetics, since they regulate not only the elimination but also the absorption and distribution of xenobiotics. The solute carrier (SLC) and ATP-binding cassette (ABC) are the most studied transporter superfamilies [44,45]. They have a major role in homeostasis by regulating transport between body fluid compartments. For this reason, these transporters are most expressed in epithelial barriers, including the liver, intestine, kidney, and the blood–brain barrier. Those families of transporters are mainly known for their relevance in drug disposal, but they also mediate the transport of endogenous substrates. It is also known that the two superfamilies share several substrates, which makes it difficult to determine a drug pathway. For example, SLC22A2, besides being the transporter of metformin, is also a prostagladin transporter; statin is a substrate for SLC22A8 (OAT3) and ABCG2 (BCRP), and can also interact with other transporters from the SLCO family. In the kidneys, OAT1-4 exhibits similar endogenous substrates, such as cyclic nucleotides (cAMP, cGMP), folate, prostaglandin E2, and α-ketoglutarate, as well as antiviral, antibiotics, and diuretics, but no steroidal anti-inflammatory drugs [45,46,47]. The complexity of unraveling the interactions between substrates, DME, transporters, and other metabolic pathways makes it crucial to study these factors, in order to improve drug treatment in patients with CKD.

## 3. Alterations of Drug Metabolism during Chronic Kidney Disease

It is now established that the pharmacokinetic disposition of drugs in patients with CKD is altered. Renal failure alters the *milieu interieur* homeostasis, with an accumulation of uremic toxins altering the renal and non-renal clearance of drugs. This makes dose adjustment challenging in these patients, with frequent drug-related adverse events. However, the knowledge of the cellular and molecular mechanisms involved is not still clearly identified. In the following section, we will expose the knowledge of the changes in DME, as well a stransporter expression and activities during CKD in animal modelsand patients. Then we will propose possible pathways to partly explain drug impairment during CKD.

### 3.1. What Do We Learn from Patients and Animals Models of Chronic Kidney Disease?

Studies in humans are not so clear, due to the complexity ofevaluating a drug pathway and each factor capable of interacting and modifying the drug metabolism. Diabetic patients with different degrees of renal impairments were treated with repaglinide, an antidiabetic agent metabolized by the enzyme CYP3A4, or by conjugation with glucuronic acid and excreted via the bile [48]. Patients with severe renal dysfunction showed a different elimination rate and a need for drug dosing adjustments [49]. Reboxetine, a selective norepinephrine reuptake inhibitor, was administrated to patients with different severity degrees of the renal disease. An increase in renal dysfunction severity was associated with increased exposure and a reduced renal clearance of reboxetine [50]. In another study, it was shown that uremia can modify the disposition of drugs undergoing a high metabolism in the liver by alterations in kidney and liver function, as well as plasma protein concentrations [51]. Patients with more severe kidney impairment needed lower doses of warfarin, a vitamin K antagonist [52]. Although the new, nonvitamin K antagonist oral anticoagulants (NOACs) seem to be beneficial in patients with renal dysfunction [53], vitamin K antagonists remain highly prescribed oral anticoagulants in this population. In another study, fexofenadine was used as a probe of drug clearance by the intestinal and liver transporters P-gp and OATP. It was shown a decreased clearance of fexofenadine with no observed alterations in CYP3A function in patients with end-stage renal disease [54]. On the other hand, using the erythromycin breath test to evaluate the hepatic activity of CYP3A4, this enzyme function was found to be reduced in patients with CKD [55].

The erythromycin breath test illustrates the difficulties to extrapolate results obtained with a given drug after its administration in terms of enzymatic and transporter activities. Indeed, it is a standard test used to evaluate the extent of CYP3A4 activity. After injection, erythromycin (labeled with carbon-14), is converted to C-14 formaldehyde par CYP3A4, then to C-14 CO_2_, which is excreted by the lungs. The amount of C-14 CO_2_ exhaled by patients is thought to reflect CYP3A4 activity. However, in fact, this test was lately shown to be also related to the activities of both an uptake organic anion transporter and the efflux hepatic P-gp transporter, responsible for the uptake and exit of erythromycin and metabolites by the hepatocytes [56]. These misinterpretations point to the need to identify all the molecular partners encountered by a given drug, to understand truly what happens after it enters the body. In this way, in vitro work could be useful.

In nephrectomized rats, only DME and efflux transporters have been studied.Phase I enzymes display altered expression. Reduced expression and activity of *CYP1A1* and *CYP3A2* (rat ortholog of the human gene *CYP3A4*) was found in the intestine [57], as well as a reduced protein expression and activity of CYP3A1, CYP3A2, and CYP2C11 in the liver [58,59,60,61]. Liver and intestinal protein levels of CYP1A2 increased in 5/6 nephrectomized Sprague–Dawley rats by 55% and 168%, respectively [62]. Phase II enzymes have also been shown to be affected during CKD. Reduced expression and activity of the acetyltransferases Nat1 and Nat2 was found in liver from rats with CKD [63]. CKD also induced changes in efflux transporters. Intestinal expression and activity of P-glycoprotein (P-gp; *ABCB1*) and MRP2 (*ABCC2*) were found to be decreased in rats after nephrectomy [64]. In the liver, contradictory results were found: P-gp was increased, with no change on MRP2 expression [65], while another team has shown no effect on P-gpexpression and an increased MRP2 protein level [66].

### 3.2. Proposed Factors Involved in Drug Metabolism Impairments in Chronic Kidney Disease

General processes specific to CKD, such as a reduction in renal excretion, moderate acidosis, and decreased protein serum level play an important role in drug disposition and clearance. However, all molecular mechanisms involved are still not well elucidated. What are the changes in expression or activities of the endogenous molecular partners involved, and which mediators are responsible for these changes? Uremic toxins that accumulate during CKD are putative factors. Since some of uremic toxins are activators of the transcription factor, aryl hydrocarbon receptor (AhR), we propose here that AhR could participate in pharmacokinetic modifications found in patients with CKD.

#### 3.2.1. Uremic Toxins

CKD induces the accumulation, in blood and tissues, of numerous solutes. Those with a negative impact on biological functions are called uremic toxins. To date, at least 185 substances have been identified and classified according to their physico-chemical characteristics and their behavior during dialysis: (a) toxins of low molecular weight (<500 Da) and soluble in water, which are well purified; (b) medium-sized toxins (≥500 Da) that are less well purified; and (c) toxins bound to proteins. These last ones, due to their fixation to proteins, are poorly eliminated by conventional dialysis techniques [67,68,69,70,71] (http://www.uremic-toxins.org/). Uremic toxins are involved in the progression of kidney disease and in the increased mortality in this population [72,73,74,75].

In the world of uremic toxins, those derived from tryptophan, an essential amino acid supplied by the diet, are of prime importance because of their associations with CKD complications and mortality [76,77,78,79,80,81,82,83,84,85,86]. Tryptophan can be metabolized through the indolic pathway by the action of intestinal bacteria. The produced indoles join the liver, to be hydroxylated by the hepatic cytocrome CYP2E1, and then indoxyl is metabolized into indoxyl sulfate (IS) by the sulfotransferase SULT1A1, into indoxyl-beta-D-glucuronide (IG) in the liver, and into indole-3-acetic acid (IAA) in the intestine and tissues [78]. Tryptophan that is not used in protein synthesis is mostly metabolized in the kynurenine pathways by tryptophan-2,3 dioxygenase (TDO) or indolamine-2,3 dioxygenase (IDO) to produce the first metabolite of this pathway, kynurenine, which is then transformed to 3-hydroxyanthranilic acid, 3-hydroxykynurenine, kynurenic acid, anthranilic acid, and quinolinic acid [78,87]. Their levels are increased during CKD; among these toxins, IS, IAA, kynurenic acid, and quinolinic acid are protein-bound.

In vitro studies suggest that uremic serum and toxins are able to impair all phases of drug metabolism: first, the cellular uptake; second, the enzymatic metabolism by phase I and phase II enzymes; and third, the extrusion from the cells. 

Reyes et al. show that uremic toxins and human uremic serum reduced the hepatic uptake transport of different drugs, such as losartan, eprosartan and propranolol [88]. Quinolinic acid or indole-3-acetic acid alone inhibits the uptake of eprosartan. The uptake of losartan into rat and human primary hepatocytes was decreased in the presence of serum from hemodialyzed patients [88]. By using HEK293 cells transfected with plasmid encoding for OATP1B1, OATP1B3, or OATP2B1, the authors show that IAA partly inhibits transport by OATP1B1, whereas indoxyl sulfate and 3-carboxy-4-methyl-5-propyl-2-furanpropionate (CMPF) inhibit transport by OATP1B3 and OATP2B1 [88,89,90]. In rat hepatocytes, IS and CMPF can directly inhibit the uptake of erythromycine by rat organic anion transporter polypeptides 2 (rOatp2) and human OATP-C transporters [91]. In addition, four uremic toxins, including IS, IAA, and kynurenic acid, directly inhibit transport by OATP1B1 and OATP1B3 in a concentration-dependent manner [90,91,92]. OATP1B1/B3 are considered as the major hepatic OATPs involved in the uptake of numerous drugs, including mainly cholesterol-lowering drugs (statins, Ezetimibe glucuronide), endothelin receptor antagonists (Bosentan, Ambrisentan), antibiotics (Rifampin), anticancer drugs (Methotrexate, Docetaxel, Atrasentan), antidiabetic dugs (Répaglinide), and antiviral drugs (Grazoprevir, Paritaprevir, Voxilaprev) (Mcfeeely). In addition, kynurenine has also been shown to be transported into the cell by LAT1, which is highly expressed in glial, neural, renal, and endothelial cells of the blood–brain barrier, where it handles L-DOPA and Melphalan [93]. Together, these data highlight the fact that uremic toxins could compete in this way with drugs to be transported into the cells.

When rat hepatocytes were incubated with human uremic serum, reduced mRNA expression of *CYP1A2*, *2C11*, *2D1*/*D2*, *3A2*, and *4A1*/*A3* was found, as well as lower activity of CYP3A and CYP1A [94]. In another work, normal rat hepatocytes incubated with uremic serum showed decreased mRNA and protein expression of CYP2C6, 2C11, 3A1, and 3A2, and decreased *N*-demethylation of erythromycin [95]. IS induces the mRNA expression and activity of CYP1A2 [96], but partly inhibits CYP3A4 metabolism [89] in primary rat hepatocytes, UTG1A1/4 activities in human liver microsomes, and mRNA expression of CYP1A7 in primary human hepatocytes [97]. In addition, a pool of uremic toxins shows a more pronounced effect on CYP and UTG activities than when they are used individually, suggesting that they are able to heighten each other’s effects. Indole produced by the microbiota from dietary tryptophan is hydroxylated by CP2E1, and could be a competitor for drugs metabolized by this cytochrome, such as anesthetics, acetaminophen, and some antiepileptic drugs.

MRP4 (ABCC4) and BCRP (ABCG2)-mediated efflux were inhibited by uremic toxins, including IS andIAA in a concentration-dependent manner, and these inhibitions could occur at concentrations found in patients [98,99]. In addition, kynurenic acid has been identified as a novel substrate for MRP4 and BCRP, and is able to inhibit BCRP-mediated transport. In accordance with these last findings, the authors found that in mice invalidated for *Mrp4* or *Bcrp*, plasma levels of kynurenic acid were increased [98]. IS decreased the expression of ABCG1 in macrophages [100], and it directly suppressed the renal expression of SLCO4C1 [101]. Since IS, IAA, kynurenine, and CMPF are transported by OAT1 and OAT3 in the kidneys, they are also capable of limiting the excretion of other substrates by directly competition [102,103]. In their work to understand changes in bile acid pool, Weigand et al. show that kynurenic acid inhibits the activity of the bile salt efflux pump, whereas the uremic toxins p-cresyl glucoronide and hippuric acid increase the activities of multidrug resistance associated proteins [99].

AST-120 is an absorbent oral charcoal, used as treatment for patients with CKD in Japan, Korea, and the Philippines. It binds metabolites with molecular weights less than 1000, reducing their absorption through the intestinal barrier [104,105] and slowing the progression of CKD. However, in patients with moderate to severe CKD, Schulman et al. could not demonstrate a slowdown in the progression of renal disease [106]. The compliance of patients to the high number of pills and the absorption of drugs withAST-120 could partly explain the absence of benefits observed in this trial [106,107]. Nevertheless, it has been shown that AST-120 reduces the uptake of two uremic toxins: IS and p-cresol sulfate (PCS). In the kidneys, the use of AST-120 in CKD rats prevented the down regulation of OAT1 transporter [108], which is also responsible for the excretion of this uremic toxin. AST-120also recovered SLCO4C1 expression in the kidneys, while decreased plasma levels of IS were observed [101]. Studies have also shown that changes on drug metabolism can be improved after hemodialysis or kidney transplantation [109,110]. Although the useof AST-120 remains still controversial, all of these studies are largely in favor of a role of circulating factors on drug metabolism.

Besides being able to affect drug metabolism acting on biotransformation and elimination phases, uremic toxins can also change a drug’s pharmacokinetics through binding to proteins. It is known that CKD affects the protein binding of several drugs [111,112]. Free, unbound fractions of the drug are usually increased during CKD due to hypoalbuminemia, a common consequence of nephrotic syndrome and malnutrition. Furthermore, uremia leads to the carbamylation of albumin, altering its binding affinity to drugs. It has been shown that uremic toxins with a high-affinity binding to albumin (IS, CMPF, IAA, and PCS) directly compete for these binding-sites with other substrates [113,114]. This competition leads to impaired distribution and an increased accumulation of the drug. This corroborates the importance of circulating molecules, such as uremic toxins, in pharmacokinetic impairments found in patients with CKD, as well as the need to improve their removal from patients. In this way, a very interesting work was recently developed by Li et al. [115]. Natural products of two plants (*Salvia miltiorrhiza* and *Carthamus tinctorius*) constituting the phytoproduct Danhong injection, used in Chinese phytomedicine, are known to bind proteins strongly. Ingeniously, the authors used the Danhong injection as a competitor of protein-bound uremic toxins, in order to increase the free fraction of these toxins and to improve their removal during a dialysis session. The results obtained are better than those observed with other treatments, such as AST-120. Indeed, the elimination is increased by 272% for PCS and by 136% for IS [115]. In addition, one could think that this natural treatment may display fewer side effects than AST-120.

#### 3.2.2. Aryl Hydrocarbon Receptor

**Figure 1:**
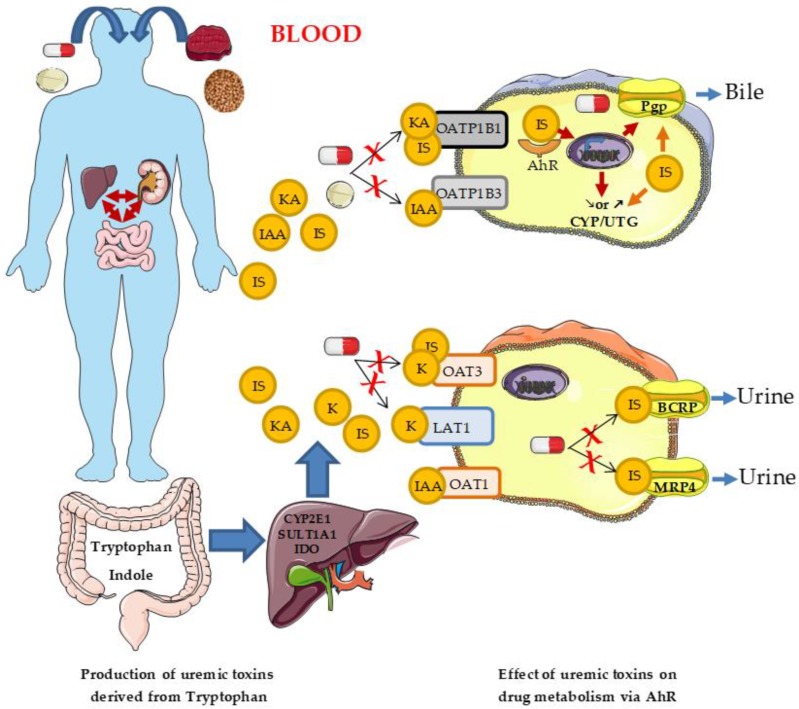
The effect of uremic toxins derived from tryptophan on drug metabolism via aryl hydrocarbon receptor(AhR). Indoxyl sulfate (IS), kynurenine (K), kynurenic acid (KA), and indole-3-acetic acid (IAA), produced from dietary tryptophan, are ligands of the transcription factor AhR. AhR, upon activation by these uremic toxins, can modulate the expression of genes involved in the metabolism and in the efflux of drugs. In addition, these toxins can inhibit directly these enzymes and these transporters.

AhR could participate in the altered clearance of drugs in patients with CKD. Besides directly disturbing enzymes, transporters, and the binding to albumin, another way for tryptophan-derived uremic toxins to modify drug metabolism is through the activation of transcription factors (Figure 1). Indeed, most of the metabolites produced during the breakdown of tryptophan by the indolic and kynurenine pathways (IS, IAA, IG, kynurenine, and kynurenic acid) are ligands of the aryl hydrocarbon receptor (AhR) [83,116,117,118]; kynurenine and kynurenic acid were recently identified as such [87]. In line with the presence of high concentrations of numerous AhR ligands in patients with CKD, AhR activation is also observed in these patients. By using a reporter gene-based cell bioassay (Callux essay), one study demonstrates that the serum of 20 hemodialyzed patients can increase by three-fold the expression of a reporter gene under the control of AhR [81]. We find same results with the serum of 116 patients with CKD [119]. In addition, by gene expression profiling from blood, we show that mRNA levels of two markers of AhR activation, CYP1A1 and AhR repressor (AHRR), are increased in 20 patients with CKD [119]. The level of AhR activation is correlated with IS levels in patients with CKD, and with kynurenine levels in non-CKD patients with post–vascular injury thrombosis [120]. In animal models of CKD (5/6 nephrectomy), AhR activation occurs as well. The mRNA level of AhRR, a specific AhR target, is increased in the kidney of CKD rats (Nakayama). Similarly, in mice with CKD, we found the induction of Cyp1a1 mRNA, a specific target of AhR, in the aorta and heart, induction which does not happen in AhR^-/-^ CKD mice. After serial indoxyl sulfate injections, we observed the expression of Cyp1a1 mRNA in the aorta and heart in WT mice, but not in AhR^-/-^ mice. These works suggest that overactivation of AhR occurs during CKD.

One of the biological roles of AhR is to respond to xenobiotic exposure, in order to orchestrate the detoxification of its ligands [120,121]. This transcription factor is sequestrated in a complex in the cytoplasm. Ligand binding to AhR induces its translocation to the nucleus [121,122] and dimerization with the aryl hydrocarbon receptor nuclear translocator (ARNT) [123]. Then, AHR/ARNT binds to a consensus DNA sequence(5′-GCGTG-3′) called axenobiotic responsive element (XRE) [124]. The binding to the promoter of target genes leads to their transcription. However, little is known about the importance of AhR on the regulation of DME and drug transporters. We performed a research in PubMed (https://www.ncbi.nlm.nih.gov/pubmed) to identify, among the unambiguous target genes modulated by AhR, those thatare involved in drug metabolism. Four of them—cytochrome 1A2 (CYP1A2), CYP2S1, aldehyde dehydrogenase 1A1 (ALDH1A1), and ALDH3A1—belong to phase I enzymes. Among phase II DME, seven are targeted by AhR: NAD(P)H quinone dehydrogenase 1 [Nqo1],UDP-glucuronosyltransferase 1A1 [UGT1A1], UGT1A3, UGT1A4, UGT1A7, UGT1A10, and glutathione S-transferase A1 [GSTA1] [35,125,126,127,128,129,130,131,132,133]. Four drug transporters—multidrug resistance-associated protein 4 (MRP4, *ABCC4*), breast cancer resistance protein (BCRP; *ABCG2*), P-glycoprotein (MDR1, P-gp, *ABCB1*), and L-type amino acid transporter 1 (LAT1)—were also shown to be regulated by AhR [134,135,136,137].

CYP1A2, whose activity is monitored by its probes—caffeine and theophylline—is among the major CYPs in the liver, and accounts for about 13% of the total CYP proteins in this organ [138,139]. In regard to the top 200 drugs, CYP1A2 is involved significantlywithat least 13 of them, and in a minor way with 110 others. Sindhu et al. demonstrated that CYP1A2 protein levels are increased by 55% and 168% in the liver and kidneys of 5/6 nephrectomized rats, respectively [46]. In the contrary, in the model of CKD induced by adenine, CY1A2 activity is not significantly affected [140]. CYP2S1 was recently discovered, and it is expressed in the skin and the small intestines. This enzyme is involved in all-transretinoic acid metabolism into the skin [141,142]. There is no information about its expression in patients with CKD. CYP1A1 and CYP1B1, which belong to the classical AhR targets, do not seem to act mainly on drugs, but rather on polycyclic aromatic hydrocarbon (PAH) metabolism [143]. Nqo1 catalyzes the transformation of the highly toxic and prooxidant quinones into hydroquinones; these are less toxic and more easily excreted by the organism. Drugs like mitomycinC, β-lapachone, and benzoquinone ansamycins are activated by Noq1 [144]. However, in another way, the increased activity of this enzyme induces resistance to 4-hydroperoxycyclophosphamide, a drug used in cancer treatment. ALDH1A1 is an important biomarker for cancer cells and cancer stem cells, and together with ALDH3A1confers resistance to oxazaphosphorines, which include cyclophosphamide in various tumor types [145,146]. Among the superfamily of UGT enzymes, UGT1 is strongly engaged in the metabolism of drugs used to treat epilepsy (UGT1A4) [147,148], schizophrenia (UGT1A3 and UGT1A4) [149], hypertension (UGT1A1, UGT1A3, UGT1A7, and UGT1A9) [150,151,152], and hypercholesterolemia (UGT1A1 and UGT1A3) [153,154,155,156]. Furthermore, UGT1A9 is responsible for the metabolization of propofol (70%) into propofol glucuronide [157]. In small-cell lung cancer cells treated by doxorubicin, GSTA1 transfection has also been shown to render these cells more resistant to doxorubicin-induced apoptosis [158].

About the drug transporters, MRP4, BCRP, and P-gp are increased upon AhR activation. They belong to the ABC-binding cassette transporters, which extrude from the cell a wide variety of chemical exogenous compounds, in order to fulfill detoxification functions and protect the organism against their toxic effects [159,160,161]. For example, several toxic compounds from plants are extruded from cells by these multidrug transporters. The alkaloid colchicine (from *Colchicum autumnale*) binds tubulin and inhibits the polymerization dynamics of microtubules. The taxane paclitaxel (from *Taxus baccata*) stabilizes microtubules by binding to polymeric tubulin. The resulting effect of these two molecules is to stop cell mitosis. Pheophorbide a, found in algae and plants, induces phototoxicity. Thus, these multidrug transporters were and are highly precious to preserve the hunter-gatherers that we were in the past. While these transporters are used to extrude mainly chemical compounds derived from plants, their very wide specificity lead them to handle drugs, and to be considered now as the main players of the multidrug resistance process (MDR) [148,162]. Extruding drugs from cells decreases their intestinal absorption, their intracellular concentration, and thus their cellular effect. Mutsaers et al. demonstrated that uremic toxins decreased some drug transporter activities [92], and could in this way counteract increased expression under AhR activation. In addition, we have found that cardiac and kidney transplant recipients with CKD needed higher doses of cyclosporine, a P-gp substrate, to obtain the cyclosporine target blood level. These in vivo results are in accord with our findings showing that IS increases P-gp activity in an AhR-dependant way in hepatocytes [137]. Among the transporter systems expressed at the blood–brain barrier (BBB), those responsible for the transport of large amino acids (large neutral amino acid transporter 1 (LAT1)) are highly expressed. These transporters could compromise drug delivery to the brain [163]. LAT1 is expressed in both the luminal and abluminal membrane of the capillary endothelial cells at the BBB, with expression levels approximately 100-fold greater than in other tissues (e.g., placenta, retina, gut).There is some evidence that supports the involvement of the LAT system on the pharmacokinetics of several drugs and hormones, such as l-DOPA, the antitumoral drug Melphalan, and in to a lesser extent thyroxine and triiodothyronine [164]. In vitro studies with the brain capillary endothelium [165,166,167,168,169], renal epithelial cells [170], intestinal epithelial cell lines [171], and plasmacytoma cells have demonstrated clearly that the uptake of l-DOPA and Melphalan takes place by L-type amino acid transporters (LAT1 and LAT2).

## 4. Conclusions

In conclusion, AhR, by its target genes, seems to play a role in drug metabolism, and could constitute a link between CKD and altered drug clearance. The modulation of its activity could be proposed as a possible and future potential target to modulate drug clearance in patients. Its level of activation in vivo, evaluated by a Callux assay, could help clinicians to adjust the dosage of some drugs. Further works are necessary to prove the usefulness of this approach in the care of patients with CKD.

## Figures and Tables

**Table 1:** Listof the drugs used for the treatment of chronic kidney disease (CKD) and CKD-related diseases.

**Cardiovascular Drugs**	**Metabolism**
β-receptor blockers	insulin
calcium channel blockers	antidiabetics (sulfonylureas, biguanides/metformin, thiazolidinediones, insulin)
antianginals	thyroid hormones
angiotensin-converting-enzyme inhibitors	diphosphonate
angiotensin receptor blockers	vasopressin analogues
Alpha blockers	calcium
thiazide diuretics	allopurinol
loop diuretics	phosphate chelators
heparin	
Warfarin	
direct oral anticoagulant	
antiplatelet drugs	
3-hydroxy-3-methylglutaryl-CoA reductase reductase inhibitors	
**Digestive System**	**For Infections and Infestations**
antacids	antibiotics
proton pump inhibitors (PPIs)	Anti-tuberculous drugs
H2-receptor antagonists	antivirals
Laxatives	
**Central Nervous System**	**For the Immune System**
hypnotics	vaccines
anesthetics	immunoglobulins
antidepressants (including tricyclic antidepressants, selective serotonin reuptake inhibitors (SSRIs) anticonvulsants/antiepileptics	immunosuppressants
benzodiazepines	monoclonal antibodies
antihistamines	corticosteroids
**Pain Killers**	
Paracetamol	
Tramadol	
pioids	
